# Keynote 1: Chris GidlowThinking about inclusion - learning from physical activity research and beyond

**DOI:** 10.1093/eurpub/ckae114.001

**Published:** 2024-09-26

## Abstract

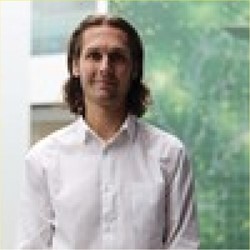

Certain individuals and population subgroups are ‘under-served’; not sufficiently included in society, health-enhancing opportunities or associated research to have the same chances of a healthy life as others. This presentation uses data from England to illustrate the reduction of public spending and worsening of the social determinants that has created conditions that exacerbate such exclusion and corresponding poor health outcomes. Specifically, learning will be shared from multiple disadvantage research, where people find themselves at the extreme margins of social disadvantage through a combination of traumatic and health-damaging life experiences (e.g., homelessness, substance misuse, mental health problems). Lessons with wider relevance to physical activity and other health promotion will be shared: the simultaneous exclusion from programmes and the research on which they are based; the critical role of lived experience to identify and understand the needs of those excluded; and applying Marmot’s concept of proportionate universalism to move from services/programmes that target equity, rather than equality, as a means of improving inclusion.

